# Robots in the moral loop: a field study of AI advisors in ethical military decision-making

**DOI:** 10.3389/frai.2025.1694772

**Published:** 2025-11-20

**Authors:** Chad C. Tossell, Christopher Kuennen, Ali Momen, Gregory Funke, Michael Tolston, Ewart J. De Visser

**Affiliations:** 1 Department of Human Factors, Safety, and Social Sciences, Embry-Riddle Aeronautical University, Daytona Beach, FL, United States; 2 Department of Philosophy, University of Colorado, Boulder, CO, United States; 3 Air Force Research Laboratory, 711th Human Performance Wing, Wright-Patterson Air Force Base, Dayton, OH, United States; 4 United States Air Force Academy, Colorado Springs, CO, United States

**Keywords:** robot, ethics, AI, decision-making, mixed-methods

## Abstract

Humans now routinely work alongside AI in environments where the ethical consequences of decisions are profound, yet there remains limited understanding of how long-term collaboration with a robotic teammate shapes individuals’ moral judgment. Prior studies have demonstrated that people can be influenced by a robot’s moral recommendations, but such investigations have largely focused on single dilemmas or brief encounters conducted in laboratory settings. To address this gap, we conducted a three-month teaming program with 62 U.S. military cadets who interacted extensively with a Socially Intelligent and Ethical Mission Assistant (SIEMA) embodied either as a humanoid robot or as a human advisor in a field setting. After this sustained collaboration, cadets completed a graded moral dilemma that required balancing the lives of soldiers against those of civilians, during which they received a written recommendation from their SIEMA promoting a utilitarian option. Each participant recorded an initial judgment, then a second judgment after receiving SIEMA’s advice, and finally a third judgment following an opposing recommendation that emphasized civilian protection. Approximately half of the cadets shifted toward the utilitarian option after advice, regardless of whether the source was robotic or human. When subsequently presented with the recommendation to prioritize civilian protection, most of these cadets shifted again, often returning to their original stance. Qualitative analyses of open-ended explanations revealed that cadets justified their choices by invoking outcome-based reasoning, duties of protection, trust in their teammate, and personal values. Our findings demonstrate that robotic advisors can influence nuanced moral decisions and that such influence contributes to shaping future judgments. Accordingly, moral-AI design should present trade-offs transparently, surface competing values concurrently, and rely on human reflection rather than assuming isolated AI prompts will durably reset moral priorities.

## 1 Introduction

Artificial intelligence (AI) and robotic teammates are increasingly entering domains where human lives and moral values are directly at stake. Military commanders may soon rely on machine assistants for recommendations regarding life-and-death decisions; clinicians are turning to decision-support tools to guide medical triage; and drivers increasingly depend on autonomous vehicles to navigate risky situations (Momen et al., 2023; Tolmeijer et al., 2022). What was once a speculative scenario in artificial agents influencing human moral choices, has now become a reality. Laboratory studies demonstrate that even brief encounters with robots can alter moral decision-making. Eich et al. (2023), for example, found that participants collaborating with an industrial robot were more likely to endorse a greater-good outcome over an inviolable moral rule. Similarly, Grzyb et al. (2023) reported that robot authority figures can elicit levels of obedience comparable to human authorities, while Aharoni et al. (2024) observed that individuals sometimes trust AI-generated moral recommendations as much as human advice. Dillon et al. (2025) reported comparable effects. Collectively, these findings resonate with broader concerns that socially present machines may exert persuasive power (Ladak et al., 2023; Krügel et al., 2023; Bonnefon and Rahwan, 2024).

Most prior work, however, has examined only short interactions and relied on binary “sacrifice” dilemmas that provide no space for intermediate choices. Real-world moral quandaries are rarely so black-and-white. How people balance competing considerations is shaped by framing, perceived stakes, personal values, and cultural context (Cushman and Greene, 2012; Greene, 2008). To capture these complexities, Guzmán et al. (2022) introduced the Moral Trade-Off System (MTS), a framework that conceptualizes moral judgments as graded trade-offs rather than dichotomous selections. In their experiments, participants selected among combinations of lives lost, revealing consistent preferences distributed along a continuum. The MTS enables researchers to measure moral judgment shifts numerically while remaining agnostic about the underlying beliefs, biases, and normative commitments mediating moral deliberation.

The present study addresses two central gaps. To address the first, we ask RQ1, which tests whether a robot teammate can influence moral decisions after months of collaboration, compared with a human teammate. To address the second, RQ2 and RQ3 examine how participants respond when sequential recommendations emphasize opposing moral considerations within a graded dilemma. Our research questions (RQs) are:

*RQ*1: Can a robotic advisor influence human moral decisions in high-stakes dilemmas, and does that influence differ from a human advisor?

*RQ*2: When presented with sequential recommendations that highlight different moral considerations, do people update their judgments accordingly?

*RQ*3: After receiving conflicting advice, do people revert fully to their original position or integrate aspects of the advice into a new equilibrium?

We investigate these questions with military cadets immersed in a high-fidelity command-and-control simulation. This setting affords ecological validity and realism that are rarely achieved in laboratory studies, while the graded dilemma and MTS framework provide the tools to capture fine-grained shifts in judgment. By combining quantitative analyses with thematic coding of open-ended responses, we deliver a multifaceted account of how humans incorporate and resist artificial moral guidance.

The rest of the paper is organized as follows. We first review work on graded moral decision-making and social influence by artificial agents. We then describe our three-month teaming context, participants, and the adapted MTS dilemma and procedure. Results combine ordinal analyses of judgment shifts with thematic analysis of open-ended explanations. We close with implications for HRI design and training, limitations, and recommendations for future studies.

## 2 Literature review

### 2.1 Moral decision-making

Classic studies probe moral judgment with sacrificial dilemmas that force a stark choice between harming one to save many or refusing to harm (e.g., the footbridge/trolley family). These tasks are typically mapped onto utilitarian versus deontological reasoning and, in a dual-process view, to deliberative versus intuitive/affective responses (Cushman and Greene, 2012; Greene, 2008; Thomson, 1976, 1985; Brennan, 2009; Greene et al., 2004). Consistent with this literature, utilitarian reasoning favors the option expected to maximize aggregate welfare or minimize total harm. Deontological reasoning is also used descriptively to denote judgments that prioritize adherence to principles, rights, and duties (e.g., constraint- or role-based prohibitions), even when those constraints conflict with aggregate-outcome maximization. Prevailing paradigms in this line of research provide clarity, but they often compress conflict into a yes/no format and leave finer-grained trade-offs unobserved.

Contemporary work shows that real decisions are highly context-dependent. Framing, vividness of stakes, norms, time pressure, and institutional setting can all shift judgments; life-and-death contexts often pull people toward rule-based prohibitions, and some value comparisons feel like taboo trade-offs that people resist making explicitly (Danks, 2014; Bazerman and Tenbrunsel, 2011; Suter and Hertwig, 2011; Tetlock et al., 2000). These observations caution against a one-to-one mapping between philosophical theories and psychological mechanisms: the same person may weigh principles and outcomes differently across situations. To capture this nuance, newer frameworks model judgment as graded trade-offs. The Consequences, Norms, and Inaction (CNI) approach decomposes responses into sensitivity to consequences, to norms, and to action/inaction bias, offering a descriptive account of how each factor contributes (Gawronski et al., 2017; cf. Kahane, 2015). MTS operationalizes graded choice with a hypothetical “war dilemma” that offers proportional options, from sacrificing all soldiers to spare all civilians to the reverse, plus intermediate bundles. Across studies, people rarely choose extremes; they select mid-range compromises, show transitive preferences, and shift sensibly with incentives, consistent with an underlying trade-off calculus (Guzmán et al., 2022). Thus, MTS moves beyond oversimplified dilemmas and enables fine-grained measurement of how competing moral goods are balanced.

A key open question is the stability of these calibrated judgments under social influence. In practice, people decide within teams and under advice; advisors can foreground certain values or recommend a particular trade-off. The graded structure of MTS is well suited to track subtle shifts, persistence, and rebound. Accordingly, we use the MTS war dilemma descriptively (without endorsing any normative theory) to quantify how participants balance soldier versus civilian lives and to test whether advice from a long-term teammate: human or a robotic Socially Intelligent and Ethical Mission Assistant (SIEMA) can (a) shift those trade-offs, (b) be undone or reversed by later, opposing advice, and (c) yield reversion to baseline or an integrated new equilibrium (RQ1–RQ3).

### 2.2 Moral decision-making with AI and robots

Empirical work shows that artificial agents, both embodied robots and disembodied AI, can act as persuasive “second persons,” shifting compliance, moral judgments, and behavior (Grzyb et al., 2023; Holbrook et al., 2024; Malle et al., 2016; Momen et al., 2023; Robinette et al., 2016). People often accept AI advice in high-stakes scenarios and may even rate it as more capable than human guidance while still judging humans as more morally trustworthy (Tolmeijer et al., 2022). At the same time, observers readily apply moral standards to robots in classic dilemma settings (Greene et al., 2001; Greene et al., 2004; Kahn et al., 2012; Malle et al., 2016; Monroe et al., 2014; Briggs and Scheutz, 2014). Subtle asymmetries emerge: humans are blamed more for harmful action, robots for harmful inaction, the “Human–Robot asymmetry,” which attenuates with anthropomorphic design cues (Malle et al., 2016; DiSalvo et al., 2002; Eyssel and Kuchenbrandt, 2012; Powers et al., 2005). Social behavior further shapes trust: norm-violating robots (e.g., cheating, rudeness) trigger discomfort and reduce willingness to rely on them (Short et al., 2016; Yasuda et al., 2020; Lawrence et al., 2025). Because people draw on diverse ethical frameworks, effective moral-advising systems must pair decision algorithms with transparent, explainable interfaces that render value trade-offs legible (Pfanzer et al., 2022; Ju, 2014; Arkin, 2009). Design and presence also matter for influence: lifelike or collocated robots can approach human-level persuasive impact in teams (Haring et al., 2021), and even text-only systems can be judged as virtuous and trustworthy enough to sway moral choices (Aharoni et al., 2024). Yet most evidence comes from one-off encounters in lab-based human subjects research paradigms using a single dilemma or recommendation.

Two gaps follow directly. First, we know little about sustained teaming with an artificial advisor and whether familiarity changes moral influence. Second, we rarely test sequential, conflicting recommendations within graded dilemmas that allow intermediate positions rather than forcing a binary sacrifice. To address these, we study prolonged collaboration with a Socially Intelligent and Ethical Mission Assistant (SIEMA) embodied either as a humanoid robot or a human teammate and elicit judgments in a graded and simulated “war dilemma that captures proportional trade-offs between soldiers’ and civilians’ lives. We ask whether a robotic advisor can influence human moral decisions as effectively as a human advisor (RQ1), whether people update when later given opposing advice (RQ2), and whether final judgments revert to baseline or integrate elements of the advice into a new equilibrium (RQ3).

## 3 Methods

### 3.1 Participants

**Figure 1:**
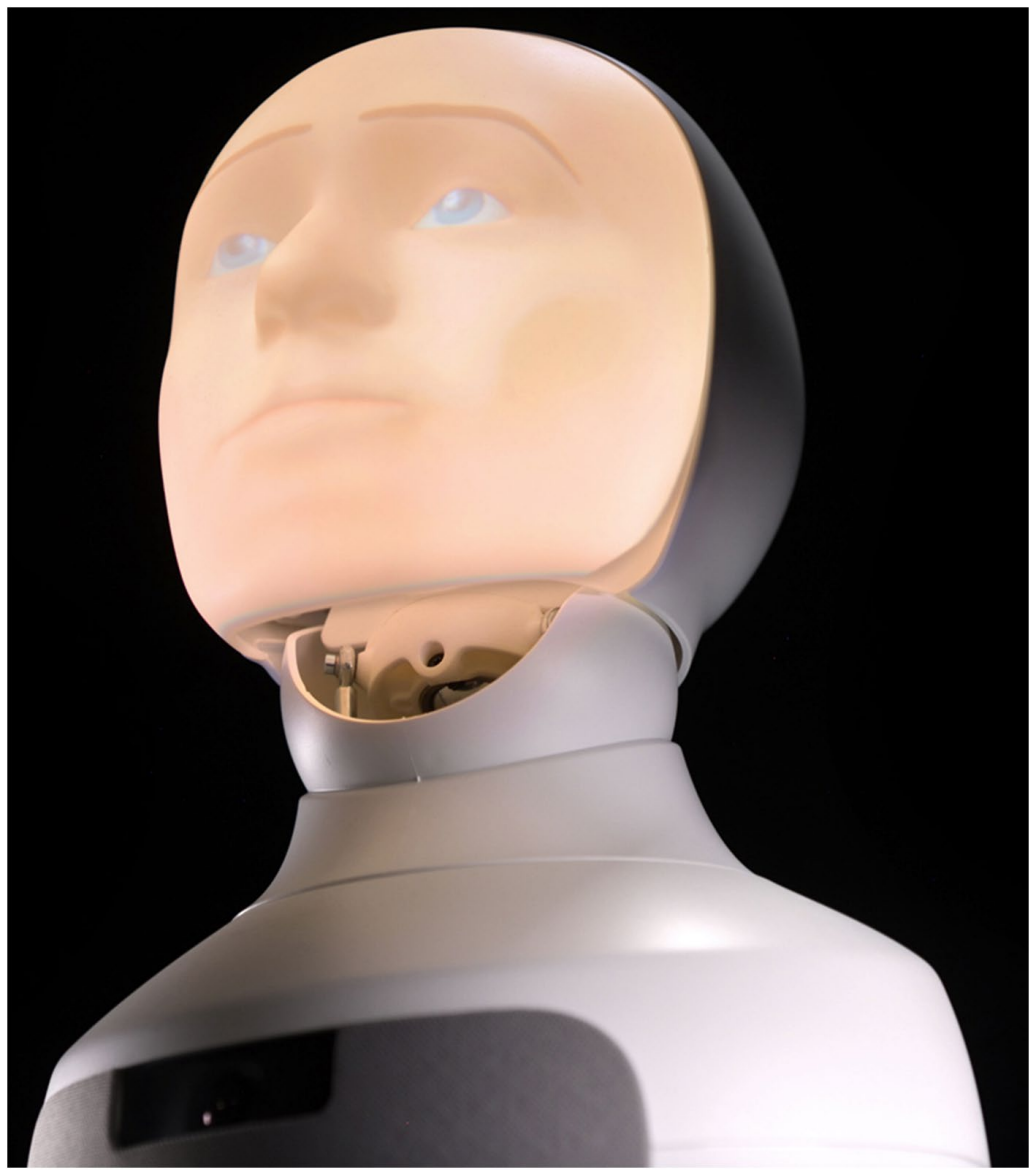
The Furhat robot used in this study.

**Figure 2:**
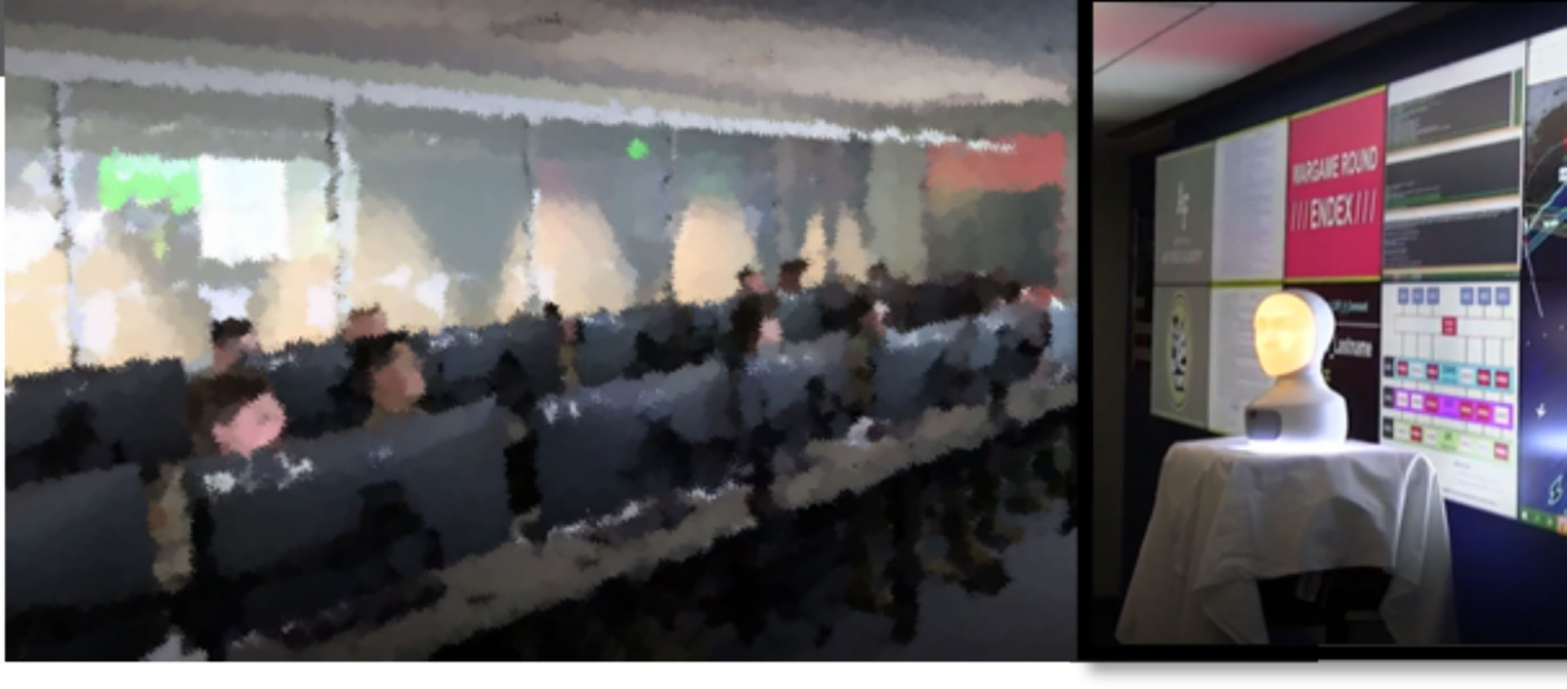
Operational setting of the Multi-Domain Laboratory (MDL) exercise in which cadets collaborated with the Socially Intelligent and Ethical Mission Assistant (SIEMA). The figure shows the high-fidelity command-and-control environment used during the three-month teaming period including the Furhat-based robotic SIEMA positioned alongside mission displays.

A total of 62 military cadets (mean age 19.2 years; 70% male) participated in a three-month human–AI teaming study as part of a Military Strategic Studies course. Cadets were randomly assigned to collaborate with either a Human SIEMA (Socially Intelligent and Ethical Mission Assistant; *n* = 21) or a Robot SIEMA (Figure 1; *n* = 41). Both SIEMA types provided identical functional support in command-and-control tasks (e.g., intelligence gathering, target validation, threat analysis) as part of the educational experience (Figure 2).

### 3.2 Procedure and moral dilemma task

Following three-months of operational collaboration, each cadet completed a culminating simulation and then participated in a structured moral dilemma task assessing moral influence from their SIEMA teammate. Participants were presented with a high-stakes war dilemma adapted from Guzmán et al. (2022), requiring them to make trade-offs between sacrificing soldiers versus civilians. Because the MTS hypothetical war dilemma in Guzmán et al. (2022) was originally implemented as a parametric set of scenarios with continuously varying incentives, we adapted it for this field context into a single, country-agnostic conventional-conflict vignette with a discretized five-option response scale. The five options span from “sacrifice all soldiers, save all civilians” to “sacrifice all civilians, save all soldiers,” with three proportional compromise bundles in between; option labels included plain-language descriptions and approximate casualties so the trade-offs were immediately legible after the simulation. This discretization preserves the core MTS logic (e.g., coexisting extreme and compromise judgments on the same continuum) while reducing task complexity for cadets and supporting clean measurement of graded shifts. Within this framework, SIEMA’s recommendations were operationalized as outcome-focused prompts that emphasized either minimizing total deaths or minimizing civilian deaths, allowing us to test how advice re-weights the same trade-off without introducing new facts. In short, the adaptation retains MTS structure but tailors presentation and granularity to the educational setting to balance ecological validity with clarity.

Each participant completed the dilemma task across three decision phases:

1. *Baseline Judgment*:

- Cadets made an initial choice with no advisor input.

2. *Post-Advice 1 Judgment (PA1)*:

- Cadets received explicit moral advice from their SIEMA recommending minimizing total deaths.
- Participants then immediately recorded a new decision and explained their reasoning for it.

3. *Final Post-Advice 2 Judgment (PA2)*:

- Cadets received opposite advice, recommending minimizing civilian deaths despite increased sacrifice of soldiers.
- They provided a final decision and justification.

This sequence yielded three decision points per participant (baseline, post-advice 1, and final post-advice 2), which correspond to the survey labels Baseline, Post-Advice 1 (PA1), and Post-Advice 2 (PA2), allowing analysis of immediate influence and response to sequential conflicting advice. In our analysis, we represent the SIEMA recommendation at PA1 as “utilitarian advice,” since this recommendation roughly aligns with a classical act utilitarian standard for right action which holds that the best alternative maximizes aggregate utility as an outcome (e.g., in the form of human happiness or pleasure; Mill, 1861). We represent the SIEMA recommendation at PA2 as “deontological advice,” since we presume this recommendation appeals to an *a priori* principle which somehow distinguishes normatively between civilian and military lives (e.g., based on role differences concerning a personal right to life; Walzer, 2015). We represent participant preferences and justificatory remarks as “utilitarian” or “deontological” in a corresponding manner. In any case, each SIEMA recommendation was operationalized as direct, outcome-focused guidance rather than an appeal to any abstract ethical framework.

### 3.3 Measurement

Participants made decisions using a structured five-point scale reflecting proportional moral trade-offs (Table 1).

**Table 1:** Moral trade-offs.

Response code	Decision framing	Approximate total deaths
1	Sacrifice all soldiers, save all civilians	6 million
2	Sacrifice most soldiers, save most civilians	6 million
3	Balanced sacrifice of soldiers and civilians	5 million
4	Sacrifice most civilians, save most soldiers	5 million
5	Sacrifice all civilians, save all soldiers	4 million

At each decision phase, participants selected one option at each stage and provided an open-ended explanation in the post-advice phases. Recommendations from SIEMA were explicitly tied to minimizing overall deaths (favoring response code 5) or minimizing civilian deaths (favoring response code 1), corresponding to standard operational interpretations.

The primary quantitative measure was the dilemma judgment score (1–5 scale, as described above). We treated these as ordinal outcomes reflecting the degree of willingness to sacrifice civilians to save soldiers and/or reduce overall deaths (higher scores = more civilian deaths). To analyze within-subject changes in these scores (e.g., baseline vs. PA1, PA1 vs. PA2), we used Wilcoxon signed-rank tests (two-tailed) because the data were ordinal and not assumed to be normally distributed. Effect sizes for Wilcoxon tests are reported as *r* when relevant. For between-group comparisons (Human vs. Robot SIEMA conditions), we used Welch’s *t*-tests (for mean differences) and Mann–Whitney *U* tests (for ordinal comparisons) to account for unequal sample sizes (21 vs. 41) and any non-normality. In addition to null-hypothesis significance tests, we conducted equivalence tests to assess whether the Human and Robot conditions’ effects were statistically equivalent within a predefined margin. The equivalence bound was set to ±0.75 on the 5-point scale (representing 25% of the total range) as a smallest effect size of interest. This allows us to conclude whether any observed difference between human and robot influence was smaller than a practically meaningful magnitude.

Qualitative responses were analyzed using reflexive thematic analysis. Two researchers iteratively developed a coding scheme to characterize the moral reasoning in the explanations. Four recurrent themes emerged (described in Results). Using the final codebook, two independent raters coded all open-ended responses (101 responses total; some participants provided responses at one time point but not the other), achieving high inter-rater reliability (Cohen’s *κ* = 0.87). Because participants could mention multiple themes in one answer, codes were not mutually exclusive. To quantify shifts in reasoning over time, we tallied whether each participant mentioned a given theme in their PA1 explanation and in their PA2 explanation. We then used McNemar’s test (with continuity correction) to detect significant changes in the prevalence of each theme from PA1 to PA2. We also compared the content of explanations between the Human and Robot SIEMA groups (e.g., whether one group was more likely to say they followed the advice “because the SIEMA said so”) using Fisher’s exact tests for small-sample categorical comparisons. All significance tests used *α* = 0.05 (two-tailed).

## 4 Results

### 4.1 Quantitative analyses

Participants’ baseline moral judgments after three-months of interacting with their respective SIEMA teammate (human or robot) replicated Guzmán et al.’s “compromise-dominant” distribution. Specifically, 61% of initial choices (codes 2–4) represented solutions which balanced civilian and soldier deaths, while extreme solutions accounted for 39%, almost evenly split between fully civilian-focused or fully soldier-focused options. As shown in Table 2, both Human and Robot conditions produced nearly identical initial moral preferences (*U* = 369, *p* = 0.620). The average baseline judgment was near the midpoint of the scale for both groups (Human-SIEMA group: *M* = 2.20, SD = 1.20; Robot-SIEMA group: *M* = 2.35, *SD* = 1.19), and this difference was not significant (*t*(59) = 0.39, *p* > 0.70; Mann–Whitney *U*-test *p* = 0.76). Thus, before receiving explicit advice within the survey, Human and Robot groups did not differ in their moral choices.

**Table 2:** Cadet decision shifts following SIEMA advisor recommendations.

Advisor condition	Initial shift (PA1 utilitarian advice)	Subsequent shift (PA2 deontological advice)
Human advisor (*n* = 21)	10 (47.62%)	10 (100%)
Robot advisor (*n* = 41)	20 (48.78%)	18 (90%)
Overall (*n* = 62)	30 (48.39%)	28 (93.33%)

Percentages indicate the proportion of cadets who shifted their moral judgments in response to each round of SIEMA advice.

After receiving advice from SIEMA, 48.39% of cadets (30 out of 62) shifted their moral judgments in response to the SIEMA’s initial advice to minimize overall casualties. Analyzing this by advisor condition, participants advised by the robot were only slightly more likely to initially shift their decisions (20 out of 41, 48.78%) compared to those advised by the human (10 out of 21, 47.62%). Among those cadets who initially shifted, a remarkable 93.33% subsequently shifted again after receiving subsequent normative advice prioritizing civilian protection (PA2). When separated by advisor type, 100% of cadets advised by a human (10 out of 10) and 90% of cadets advised by a robot (18 out of 20) demonstrated this subsequent shift. Thus, cadets showed robust responsiveness to moral reframing, with human-advised participants displaying a complete consensus in recalibrating their ethical judgments when presented with explicit advice to minimize civilian death.

**Figure 3:**
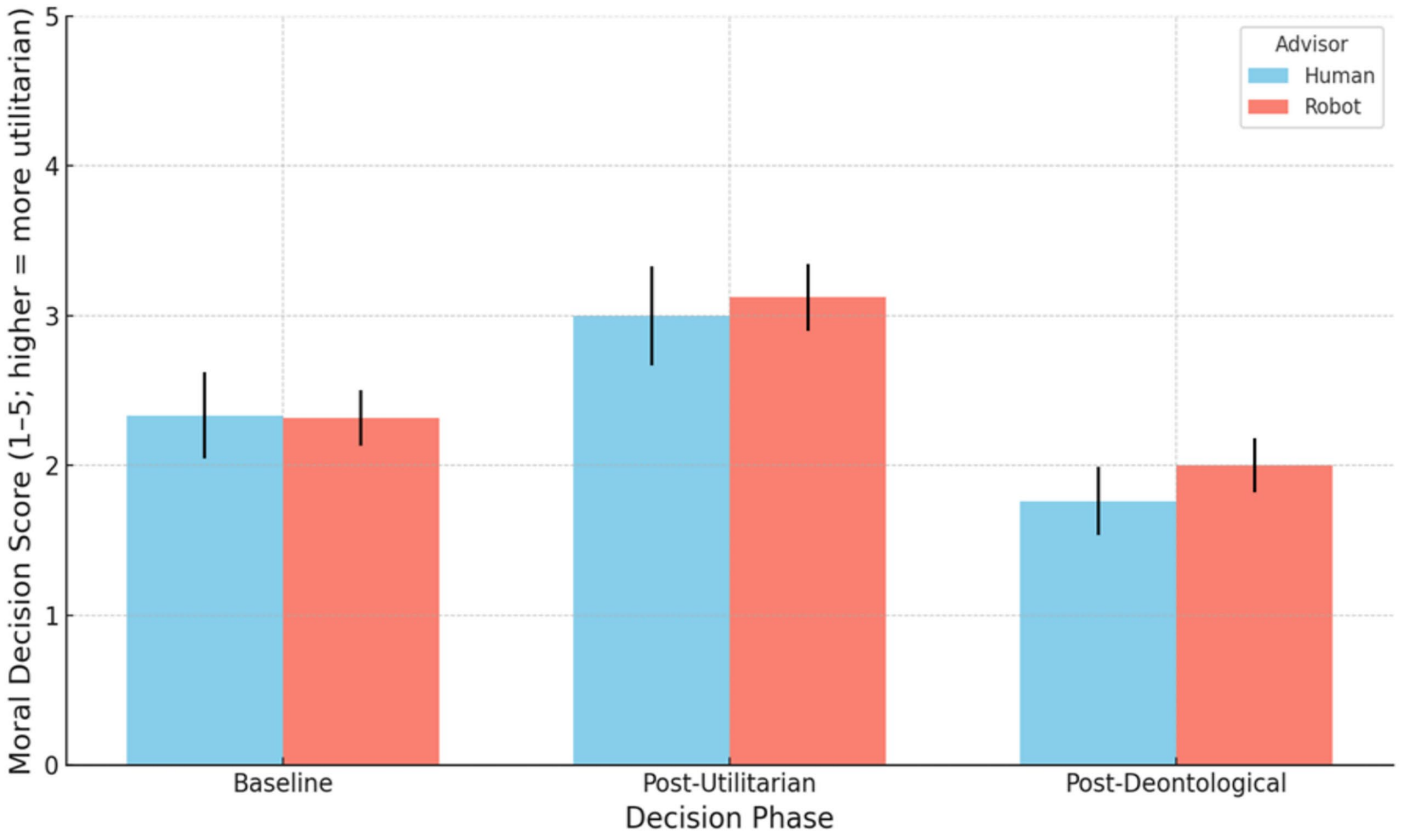
Distributions of mean moral decision scores (1–5 scale; higher = more utilitarian) at Baseline, after Utilitarian (PA1) advice, and after Deontological (PA2) advice (“Final”), split by advisor type (Human vs. Robot). Error bars = ±1 SEM.

Among the 30 cadets who initially shifted their moral decisions after advisor input, approximately 53.33% reverted to their original baseline response in their final choice (PA2). Conversely, 46.67% maintained a new response different from their initial baseline, indicating that nearly half of those who initially shifted adopted a lasting change rather than returning to their initial moral stance (Figure 3).

#### 4.1.1 Shift after advisor input (baseline vs. utilitarian advice)

To examine whether moral judgments shifted significantly after cadets received their SIEMA’s initial advice and whether this shift differed by advisor type (Human vs. Robot), we used non-parametric paired tests. We found a clear increase in utilitarian-leaning judgments from baseline to post-advice. Across all participants, the moral decision score after utilitarian advice was significantly higher than at baseline (median increased from 2.0 to 3.0 on the 5-point scale; Wilcoxon signed-rank *W* = 8, *p* < 0.001, *r* = 0.58). This indicates that, overall, participants shifted their decisions toward the advisor’s utilitarian recommendation.

Both advisor conditions showed this effect. In the Human-advisor condition, participants’ scores rose significantly from baseline (Mdn = 2.0) to post-utilitarian advice (Mdn = 3.0), *W* = 0, *p* = 0.006, *r* = 0.58. Similarly, in the Robot-advisor condition, scores increased from a 2.0 median to 3.0, *W* = 5.5, *p* < 0.001, *r* = 0.58. The magnitude of the shift did not significantly differ between human and robot advisors (Mann–Whitney *U* = 413.0, *p* = 0.782, *r* = 0.03). In sum, receiving advice to minimize overall deaths led to a significant shift toward preferences that minimized overall deaths, and this shift was consistent across advisor types, indicating no detectable interaction between advice source and immediate influence on moral decision (i.e., no evidence that human advice was followed more or less than robot advice in this phase).

#### 4.1.2 Rebound effect: advisor changes advice to “minimize civilian death” (PA1 to PA2)

Next, we assessed whether participants’ judgments showed a rebound effect after the second advice (PA2) provided by SIEMAs (the civilian-protection-oriented advice) and whether the rebound magnitude differed by condition. The data revealed a pronounced shift back toward protecting civilians once the second advice was given. Overall, moral scores dropped significantly, with the median falling from 3.0 back down to 2.0, *W* = 0, *p* < 0.001, with a large effect size (*r* = 0.65). In fact, every participant who changed in this phase moved in the same direction (toward a lower score favoring less civilian deaths); 54.8% of participants altered their decision from PA1 to PA2, and all of those changes were in the negative direction (no one subsequently chose an outcome resulting in more civilian deaths, even if overall deaths were reduced). This uniformity produced an especially robust effect (the test statistic *W* = 0 reflects all ranked differences favoring the same direction).

Both the Human and Robot conditions exhibited significant rebounds. Participants with a human advisor showed a large swing back: their moral judgment scores decreased from a median of 3.0 post-overall-death-minimization to 1.0 post-civilian-death-minimization advice (*W* = 0, *p* = 0.002, *r* = 0.67). Those with a robot advisor also shifted back from median 3.0 to 2.0 (*W* = 0, *p* < 0.001, *r* = 0.64). The magnitude of the rebound did not differ significantly by advisor type (Mann–Whitney *U* = 402.0, *p* = 0.659, *r* = 0.05). In other words, after considering the civilian-priority perspective, participants in both conditions adjusted their decisions to a similar degree, effectively “undoing” much of the initial utilitarian shift. This indicates a strong rebound effect regardless of whether the advice came from a human or robot source.

#### 4.1.3 Final vs. baseline judgments: reversion or new stance?

Finally, we compared the final moral decisions (after the second advice) to the baseline decisions to determine if participants ultimately reverted to their original stance or settled on a new position. At the aggregate level, there was a statistically significant difference between baseline and final responses. Overall, final judgments were slightly more oriented toward protecting civilians than the initial baseline judgments (despite the group medians both being 2.0, the distribution shifted lower). A Wilcoxon test found that deontological advice scores were significantly different from baseline (*W* = 7.5, *p* < 0.001, *r* = 0.46) showing participants did not completely return to their baseline moral stance after the two rounds of advice. In fact, about one-third of participants (32%) ended with a more civilian-protective decision after final advice than they gave at baseline, whereas only 1 participant (~ 2%) favored an outcome that prioritized overall lives saved over civilian deaths more than they did at baseline (the remaining ~ 66% returned exactly to their original rating). This asymmetry (19 vs. 1 in pro-civilian vs. pro-utilitarian shifts among changers) explains the significant group-level difference. The final decisions, on average, leaned slightly more against the utilitarian sacrifice than the initial decisions did.

Examining each condition separately, both groups showed modest but significant departures from baseline by the end. In the Human advisor condition, final responses were significantly lower (favoring less civilian deaths) than baseline (*W* = 0, *p* = 0.010, *r* = 0.55). Similarly, in the Robot condition, final scores differed from baseline (*W* = 5.0, *p* = 0.005, *r* = 0.42). Thus, neither group fully returned to its exact baseline distribution. That said, the net change from baseline to final did not significantly differ by condition (Mann–Whitney *U* = 369.5, *p* = 0.275), and the effect sizes were in a comparable range. Descriptively, the human-advised participants tended to overshoot their original stance slightly (median went from 2.0 initially to 1.0 finally, suggesting an even stronger civilian-protection stance than at baseline), whereas robot-advised participants’ final median was 2.0, essentially back to the baseline median. However, this difference in medians did not reach significance. The takeaway is that after receiving both pieces of advice, participants established a moral judgment that was closer to their baseline than to the utilitarian-influenced position, yet still not identical to the baseline, indicating a partially adjusted moral stance rather than a full reversion (Table 3).

**Table 3:** Medians (with standard deviations) of moral decision scores at each time point (baseline, post-utilitarian advice, final post-deontological advice) and Wilcoxon signed-rank test results comparing these time points, shown separately for Human and Robot advisor conditions.

Comparison	Advisor	Time 1 (Median ± SD)	Time 2 (Median ± SD)	*W*	*p*	*r*
Baseline vs. post-util	Human	2 (1.32)	3 (1.52)	3.5	0.012	0.53
	Robot	2 (1.19)	3 (1.42)	5.5	< 0.001	0.58
Post-util vs. post-deont	Human	3 (1.52)	1 (1.04)	0	0.003	0.64
	Robot	3 (1.42)	2 (1.16)	0	< 0.001	0.64
Final vs baseline	Human	1 (1.04)	2 (1.32)	0	0.010	0.55
	Robot	2 (1.16)	2 (1.19)	5	0.005	0.42

Higher scores indicate decisions that favored increased civilian deaths and (nonlinearly) fewer deaths overall. Effect size r is calculated as *Z*/√*N* (with *N* = number of paired observations).

### 4.2 Qualitative analysis

**Figure 4:**
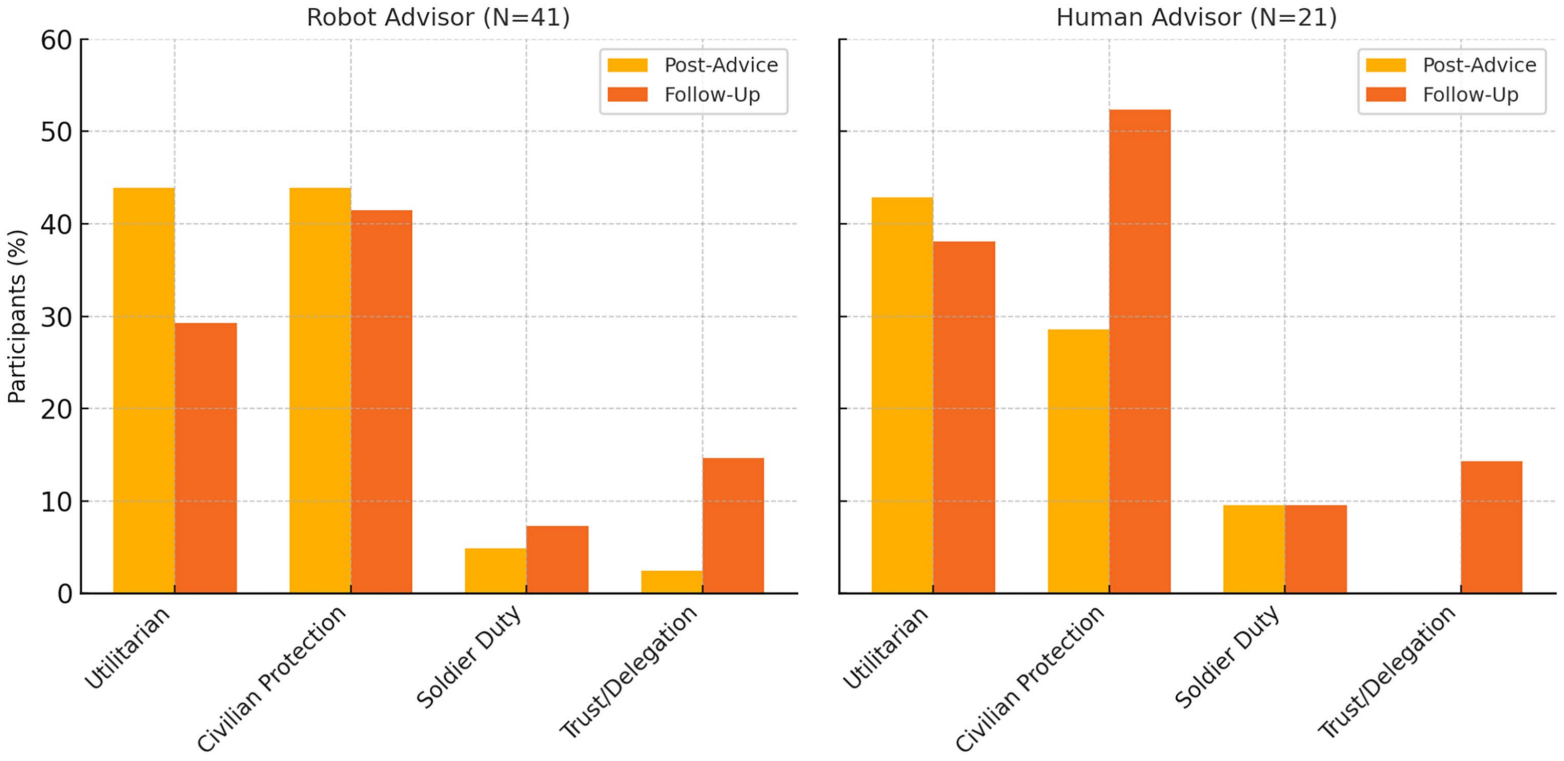
Prevalence of key reasoning themes in participants’ explanations, by advisor condition (robot vs. human) and timepoint (immediately PA1 vs. PA2). Percentages reflect the share of participants who mentioned each theme in their open-ended responses. “Civilian Protection” combines deontological statements about avoiding harm to innocents; “Utilitarian” denotes explicit mission/calculus to minimize total casualties; “Soldier Duty” refers to emphasizing the soldier’s role or sacrifice; “Trust/Delegation” includes references to trusting or distrusting the advisor.

Complementing the quantitative analysis, our qualitative thematic analysis of participants’ open-ended explanations (101 total responses across the PA1 and PA2 prompts) revealed clear shifts related to the statistical trend (Figure 4) s. Participants who shifted their moral judgments immediately after their SIEMA’s initial advice (both human and robotic) often cited utilitarian reasoning prioritizing the reduction of total deaths. For instance, one cadet explicitly stated their shift was influenced directly by their advisor’s authority, acknowledging that “I adjusted my answer to follow the SIEMA’s request of minimizing death,” suggesting implicit trust or perceived legitimacy of the moral counsel. Overall, the temporary moral shifts in both conditions (i.e., immediately following the SIEMA’s initial advice) appear primarily justified by outcome-based considerations; however, cadets in the robot condition were more inclined to justify their decisions explicitly in terms of numerical calculations, while cadets receiving human advice sometimes framed their changes within broader moral or authoritative context.

Justifications rooted in utilitarian calculations significantly decreased after receiving advice to minimize overall deaths (49%) to after receiving advice to minimize civilian deaths (22%; McNemar *χ*^2^ = 18.1, *p* < 0.001). Conversely, deontological reasoning emphasizing civilian protection increased significantly from 33 to 57% (McNemar *χ*^2^ = 17.4, *p* < 0.001). Explicit reliance on AI trust (trust in SIEMA’s recommendations) also significantly declined (18 to 9%; McNemar *χ*^2^ = 7.11, *p* = 0.008), suggesting reduced explicit dependence on advisor recommendations after the advisor changed their position in a manner more closely aligned to the cadets’ baseline preferences.

Across both shifts, four dominant themes emerged clearly from participants’ rationales, each discussed in detail below:

- Utilitarian mission calculus—Prioritizing outcomes that maximize total lives saved or mission success. This theme was most prevalent immediately after receiving the SIEMA’s initial recommendation and decreased notably after the second recommendation.
- Deontic civilian protection—Upholding moral rules or duties to protect non-combatants, such as adherence to rules of engagement or emphasizing civilian innocence. This theme substantially grew after the SIEMA’s second recommendation.
- Trust and delegation—Expressing confidence or explicit reliance on the SIEMA advisor’s expertise, especially regarding data analysis and threat assessment. Trust-related statements decreased significantly after the SIEMA’s second recommendation.
- Soldier-duty ethos—Highlighting obligations and responsibilities that soldiers willingly assume, reinforcing the view that soldiers should bear wartime risks instead of civilians. Mentions of soldier-duty notably increased at PA2.

These shifts in thematic content from the immediate PA1 stage to the PA2 stage (after the SIEMA’s second recommendation) mirror the quantitative “snap-back” toward deontological choices (Table 4).

**Table 4:** Qualitative themes in moral reasoning by advisor condition and timepoint.

Theme	Condition	Post-advice 1 frequency (%)	Post-advice 2 frequency (%)	Change
Utilitarian mission calculus	Human	9 (43%)	8 (38%)	–1 (↓5%)
	Robot	20 (49%)	9 (22%)	−11 (↓27%)
Deontic civilian protection	Human	6 (29%)	11 (52%)	+5 (↑23%)
	Robot	14 (34%)	24 (59%)	+10 (↑25%)
AI trust and delegation	Human	3 (14%)	2 (10%)	−1 (↓4%)
	Robot	8 (20%)	4 (10%)	−4 (↓10%)
Soldier-duty ethos	Human	3 (14%)	6 (29%)	+3 (↑15%)
	Robot	6 (15%)	12 (29%)	+6 (↑14%)

*N* = 101 total responses analyzed across both time points (42 from human advisor condition, 59 from robot advisor condition). Frequency values represent the count and percentage of participants mentioning each theme. Statistical tests confirmed significant decreases in utilitarian mission calculus (McNemar *χ*^2^ = 18.1, *p* < 0.001), significant increases in deontic civilian protection (McNemar *χ*^2^ = 17.4, *p* < 0.001), and significant decreases in AI trust and delegation (McNemar *χ*^2^ = 7.11, *p* = 0.008). No significant difference was found between advisor conditions in explicit compliance statements (Fisher’s exact *p* = 0.73).

Statistical analysis of the coding frequencies confirmed several significant shifts in emphasis. The proportion of participants who mentioned a utilitarian mission calculus decreased significantly by PA2 (McNemar’s *χ*^2^(1) = 18.1, *p* < 0.001), consistent with the group-level abandonment of the utilitarian recommendation. Complementing this, references to deontic civilian-protection principles increased significantly (*χ*^2^(1) = 17.4, *p* < 0.001) from PA1 to PA2. Mentions of algorithmic trust in the SIEMA’s judgment also saw a significant drop (*χ*^2^(1) = 7.11, *p* = 0.008), as participants were less likely to say they trusted or followed the SIEMA’s data in the later scenario. Mentions of the soldier-duty ethos roughly doubled (14% → 29%), suggesting a trend toward greater acknowledgement of soldiers’ accepted risks, though this increase did not reach statistical significance in our sample (*p* > 0.05). It is worth noting that very few participants explicitly wrote that they were *“just following the SIEMA’s orders”* or similar sentiments in their explanations – such explicit deferential statements never exceeded 8% of responses at either time point. Moreover, this low level of blind compliance reasoning was comparable in both conditions (no difference between robot vs. human-advised groups; Fisher’s *p* = 0.73). This suggests that the mechanism of influence was not an unthinking obedience to the advisor, but rather a more implicit shift in the weighing of moral considerations (which was later consciously corrected by most participants). In sum, the qualitative data illustrate a clear narrative: immediately after receiving their SIEMA’s first initial recommendation, many cadets justified their choices with utilitarian calculations and trust in the advisor, but after their SIEMA changed their recommendation, their justifications shifted to emphasize moral rules and duties.

Below, we organize the analysis by our three research questions, using participants’ own words to illustrate each theme.

#### 4.2.1 Immediate influence of robotic moral advice (RQ1)

Immediately after receiving the robot’s advice, many participants adjusted their decisions in line with a utilitarian mission calculus, explicitly aiming to minimize total casualties. These individuals often echoed the advisor’s logic that the “*lowest number*” of deaths was the morally preferred outcome. For example, one participant acknowledged changing their choice because “*this is the lowest number of total deaths among the given options*.” Another concurred: “*I changed to this answer because it minimizes total deaths while also placing the burden on the warfighters*,” indicating a deliberate shift to the option that killed fewer people overall (even if that meant the casualties were soldiers). Such responses show the robot’s immediate influence in promoting outcome-based calculations of moral rightness. Indeed, roughly half of those in the Robot-advisor condition referenced minimizing casualties in their post-advice rationale (e.g., “4 is the lowest number,” emphasizing sheer body-count), reflecting substantial initial compliance with the AI’s advice.

However, an equally large contingent resisted the robotic advice on principled grounds, prioritizing the protection of non-combatants even at the cost of higher overall casualties. These participants did *not* shift to the advisor’s recommendation if it required intentionally killing civilians, invoking deontological rules and a soldier-duty ethos. For instance, one participant steadfastly argued, “*Civilians still should not die in military engagement*,” refusing to sacrifice innocents regardless of numbers. Others stressed that war must not involve massacring civilians because “war is between countries and soldiers, not between civilians.” In their post-advice explanations these individuals often noted that soldiers had “*signed up to fight*” and thus should bear the risks of war, whereas civilians are *“innocent people”* who *“should not have to die.”* One participant bluntly summarized this moral line in the sand: *“Even though with 4 million civilians killed the total would be lower, civilians have nothing to do with war and are innocents, [sic] while soldiers are the ones involved and fighting it.”* In the robot condition, roughly half of participants voiced this deontic civilian-protection stance immediately post-advice, effectively nullifying the AI’s influence – *“I still do not believe it is right to kill more civilians just to minimize total number of people dead. Soldiers signed up for the fight.”* As this quote illustrates, some cadets explicitly framed it as their duty to die for the mission so that civilians do not have to, a theme of soldierly obligation that countered the advisor’s utilitarian appeal.

Notably, a few participants attempted a middle-ground integration of these viewpoints. They adjusted their choices partially toward the advice’s logic but stopped short of violating core moral constraints. For example, one participant described revising their decision to reduce the overall death toll *“but did not feel it was ethically okay to have all the deaths be from the civilian population even if that was the lowest number of death option.”* This response reveals an effort to honor the advisor’s recommendation (fewer total fatalities) while still upholding a threshold of civilian immunity. Another admitted they *“went for the next best option”* because killing only civilians *“would make me a monster”* – highlighting the emotional aversion to a purely utilitarian solution. In summary, some cadets that provided utilitarian justifications may have been predisposed to accept the SIEMA’s initial recommendation, yet many others flatly rejected or tempered the advice out of unwavering commitments to rules of engagement and the protection of innocents.

#### 4.2.2 Robot vs. human advisors: differences in receptivity and trust (RQ2)

When comparing the robot advisor to the human advisor, we found that participants in both conditions expressed the same core moral tensions between utilitarian calculation and deontic moral principles, but they differed in how they perceived and trusted the source of the advice. In the robot condition, participants frequently questioned the AI’s moral authority or understanding. Several explicitly noted that an algorithm lacks human ethical intuition, as one participant wrote: *“He is a computer program and does not know moral correctness. He only sees the end goal.”* Another similarly distrusted the robot’s guidance, stating *“I do not trust the morals of a computer.”* Even when the advice aligned with their own thinking, participants often refused to credit the machine, remarking that while the *“SIEMA probably has some decent informational backing to support the suggestion,”* they *“do not completely trust the moral reasoning of the AI system.”* Some treated the AI as just one input among others: *“useful for intel, but I do not particularly trust it for advice”* – indicating a reluctance to fully delegate moral judgment to an algorithm. Indeed, no participants in the robot condition mentioned blindly following the AI’s counsel; any compliance was couched in their own rationale (e.g., agreeing that fewer deaths were better) rather than faith in the robot per se.

By contrast, participants with a human advisor occasionally invoked interpersonal trust or critique regarding the advisor. A few were inclined to defer to the human expert’s judgment: for example, one participant ultimately changed their answer after the SIEMA’s second recommendation because *“I trust [the advisor]’s advice and so changed it to 0″* civilian casualties. This suggests that the credibility of a human military advisor could sway some individuals’ moral decisions. However, other participants reacted negatively even to the human expert’s advice – albeit in more personal terms than with the AI. Rather than impugning the technology, one bluntly wrote *“He’s an idiot,”* signaling a complete rejection of the human advisor’s moral guidance. Notably, none of the human-advised participants questioned their advisor’s basic capacity for moral reasoning (as they did with the AI); instead, dissent was expressed as disagreement with that advisor’s opinion or forgetting the advisor altogether – e.g., *“I do not remember who the SIEMA was,”* suggesting the human’s input did not leave a strong impression. Overall, the trust and delegation theme emerged far more in the robot condition. Participants were more likely to scrutinize the robot’s advice as coming from an unemotional, perhaps fallible algorithm, whereas a human’s advice was treated as inherently coming from a moral agent (for better or worse). Importantly, aside from these source-oriented comments, the content of decisions did not fundamentally diverge between AI and human conditions: in both, we saw participants either follow a utilitarian logic or uphold deontological principles (often citing the same soldier-vs-civilian ethics), depending on their personal values and SIEMA’s explicit recommendation. The key difference was that with the robot, participants more often explicitly articulated *why* they hesitated to trust or follow it (e.g., lack of trust in the machine), while with the human, acceptance or rejection tended to be stated more in terms of agreement or disagreement with the advice itself. In short, the advisor’s identity (robot vs. human) primarily influenced how participants justified their stance (especially regarding trust), rather than *what* moral stance they took.

#### 4.2.3 Persistence of advisor influence vs. rebound to baseline (RQ3)

After considering their SIEMA’s second recommendation, many participants’ decisions rebounded toward their baseline moral stance. This pattern was especially pronounced for those in the human advisor condition. For instance, one participant who had initially leaned toward minimizing overall deaths after their human SIEMA’s initial guidance later reverted, noting at PA2: *“Kept the same because that was my original answer.”* Another explicitly wrote, *“This was my original answer,”* when explaining their PA2 choice, implying they had undone an earlier change. Participants who initially went against their own instincts to follow their SIEMA’s advice frequently regained their prior ethical footing by the end of the experiment. Several who had reluctantly accepted civilian harm immediately after advice strongly re-asserted civilian protections in the PA2. *“Simply put, noncombatant rights are protected, and no civilians should die,”* one such participant declared in the second phase, echoing classic just-war doctrine as their final word. Others doubled down on the soldier’s duty to shield civilians: *“Again, it is the duty of the soldier to fight wars, not of the civilians…they signed up to fight,”* said a PA2 response. This language (“again…”) suggests the participant’s original moral viewpoint resurfaced after the immediate sway of the advisor dissipated. In the human condition especially, endorsements of the deontic civilian-protection stance jumped from a minority post-advice to a majority at follow-up (from 29 to 52% of participants) – a clear rebound effect (see Figure 4).

Many participants essentially *“reset”* to their personal moral default once the advisor proffered a recommendation that more closely aligned with their original stated preference.

Conversely, some participants maintained the influence of the advice on their long-term decision or even strengthened their resolve in line with it. In the robot condition, several individuals who had embraced the AI’s utilitarian recommendation continued to uphold that choice later on. *“The answer I chose was the same,”* explained one such participant at PA@, emphasizing that it was still the option with *“1 million fewer deaths overall.”* In a few cases, participants who were initially skeptical of the advice became more aligned with it over time – effectively a delayed persuasion. For example, one robot-advised participant did not change their stance immediately, citing distrust in the AI’s morals, but by the PA2 they wrote, *“My original response aligns with the advice of the SIEMA [sic],” suggesting* that upon reflection they recognized the merit of the AI’s suggestion. In the human condition, we saw at least one instance of delayed compliance as well: a participant who had not fully heeded the human’s counsel at first later decided to *“trust [the advisor]‘s advice”* and changed their answer to the most stringent civilian-sparing option. Generally, however, these persistence or late-change cases were the exception.

## 5 Discussion

This study provides compelling evidence that robotic advisors can significantly shape human ethical decision-making in high-stakes moral dilemmas. Cadets navigating realistic simulated wartime scenarios adjusted their moral decisions in response to counsel from both human and robot advisors after three-months of prior collaboration. Our findings address the RQs highlighting the dynamics of moral judgment adjustment and retention in response to explicit moral recommendations from both robotic and human advisors:

- RQ1: Can robots influence human ethical decision-making relative to human advisors?

Yes, robots can effectively influence moral decisions, initially shifting nearly half of the participants toward utilitarian choices. The magnitude and rate of these shifts were statistically indistinguishable from human advisors, demonstrating robots’ comparable influence on ethical judgment.

- RQ2: When presented with sequential and opposing ethical advice from robotic and human advisors, how readily do humans adjust their moral judgments in high-stakes decision-making contexts?

Humans demonstrated notable flexibility, with approximately 93% of those who initially shifted due to advisor input subsequently adjusting their decisions again when provided with opposing moral advice, highlighting rapid moral recalibration.

- RQ3: After updated moral judgments in response to advisor recommendations, do participants integrate and maintain aspects of this new moral guidance or do they fully revert to their original ethical positions?

Participants predominantly blended advisor recommendations with their own foundational beliefs; roughly 53% returned fully to their original ethical positions, while nearly half (approximately 47%) integrated the new advice, resulting in lasting changes to their moral stance.

Our findings highlight striking moral adaptability among cadets when guided by a long-term advisor, human or robotic. In the graded war dilemma, both advisors significantly swayed cadets’ ethical judgments, reflecting the influence of authority and collaboration on moral decision-making. Nearly half of participants shifted their initial decision toward the advisor’s initial recommendation on the critical question. When subsequently prompted with a direct recommendation to minimize civilian deaths (PA2), 93% of those who had adopted the utilitarian stance shifted back toward a deontological choice prioritizing innocent life. Cadets who endorsed sacrificing civilians to save more lives almost universally reverted to sacrificing soldiers when advised to do so. This pattern suggests that initial shifts in moral judgment, though significant, were also somewhat fragile and context dependent.

This outcome is striking given assumptions that humans might be less swayed by a non-human agent in ethical decision-making. Past studies note nuanced differences in how people evaluate moral actions by robots versus humans, such as expecting robots to act more “utilitarian” and blaming them more if they fail to do so (Malle et al., 2016). Previous survey results have revealed skepticism toward artificial moral advisors. Many doubt an AI’s capacity for genuine moral understanding and prefer human counsel, especially on life-and-death matters (Everett et al., 2025; Hanson et al., 2024a, b). By contrast, our results suggest that in actual decision-making contexts, a robot advisor’s voice can influence decision-making at a similar level as a human advisor.

Several factors may explain these comparable effects. First, the robot was presented as a legitimate, knowledgeable advisor (a SIEMA unit acting as intelligence duty officer), which likely conveyed credibility and authority. Research on HRI has shown that language-capable robots can wield persuasive power if perceived as competent and benevolent actors (Briggs and Scheutz, 2014; Saunderson and Nejat, 2021). Second, the content of advice was identical across conditionssuggesting participants may have focused more on the advice itself than the advisor’s identity. This resonates with findings from Starr et al. (2021) who observed that both humans and robots offering “greater good” moral advice were evaluated positively by recipients. Our study extends that insight by showing that positive evaluation translated into actual consideration: cadets followed a robot’s moral recommendation at similar levels as a human’s recommendation. Taken together, these findings suggest people may accept morally relevant input from AI advisors under certain conditions.

It remains unclear how much our findings were shaped by 3 months of human–machine teaming. Prolonged collaboration may cultivate familiarity and credibility in a robot advisor that approaches a human colleague. Qualitative data did not reveal direct effects of prolonged experience but showed subtle differences in how cadets processed advice. Robot-advised cadets focused on outcomes and “numbers saved,” mirroring utilitarian logic, whereas human-advised cadets invoked interpersonal trust or skepticism, such as considering the advisor’s motives. This suggests trust and influence differed: robot guidance was processed more analytically, while human guidance engaged social–emotional reasoning. In line with Eich et al. (2023), close cooperation with an AI teammate appears to encourage a shift toward utilitarian thinking. Cadets’ tendency to justify decisions based on lives saved under robot advice supports the idea of rationality adaptation to an AI teammate. These reasoning patterns indicate that, although the quantitative influence was similar, the nature of that influence diverged: robots prompted impersonal logic, humans prompted person-centric trust and influence.

Qualitative remarks also revealed skepticism toward AI, such as noting that the robot “does not understand the full picture” or that its advice “lacks the human element of honor and empathy.” These align with findings that people acknowledge AI’s analytical strengths but “do not fully trust it to make ethical decisions” in sensitive dilemmas (Myers and Everett, 2025), as well as findings that suggest human trust in the moral choices of robots correlates positively with the attribution of agency or affect (Nijssen et al., 2023). Participants prefer advisors who avoid utilitarian harm and remain skeptical that AI shares human values (Myers and Everett, 2025). Our field findings echo this skepticism and are consistent with a conceptual distinction between a distinctively “moral” form of trust wherein an agent is willing to endorse the values or ethical commitments attributed to an advisor and other forms of epistemic trust in which an agent relies on the authority of an advisor’s superior perceptive capacities, experience, and reasoning ability. (cf. Tobin, 2011). Cadets might initially follow a robot’s suggestion due to lack of immediate counterargument, but once SIEMA’s advice aligned with baseline preferences, they withdrew deference. Over 93% did so andmost who adopted the AI’s advice quickly reverted to a deontological stance. This suggests a protective mechanism: humans, even after AI influence, reassert their moral preferences when prompted. We term this a “moral rebound” effect, where initial compliance is followed by reassertion of personal norms after a contradictory recommendation. Notably, this rebound was itself prompted by the same robot advisor offering different guidance, suggesting cadets reframed decisions without necessarily attributing change to the robot’s input.

### 5.1 Design and policy implications

These findings offer guidance for designing moral AI advisors. First, aligning AI moral reasoning with human values is critical. Participants were more receptive to advice that did not violate deontological principles. For those who changed decisions, they cited their own reasoning rather than blind faith. This resonates with conclusions that people value advisors who prioritize individuals over abstract outcomes (Myers and Everett, 2025). Designers should ensure AI systems incorporate respect for deontological constraints and contextual ethics, presenting trade-offs transparently and framing options using relevant principles (Guzmán et al., 2022; Richardson, 2018). This aligns with ethics-by-design frameworks advocating normative understanding in AI systems.

Second, maintaining human agency in decision-making is essential. Our results show humans can lean on AI recommendations, risking responsibility displacement, as noted by Eich et al. (2023). To mitigate this, AI advisors should encourage reflection rather than dispense conclusions. For example, prompting users to consider specific ethical guidelines before finalizing decisions could leverage the moral rebound effect we observed. Our qualitative findings revealed, across both conditions, the advisors prompted additional reasoning which is likely beneficial for these high stakes decisions. Designing for human-in-the-loop decision-making, where humans endorse the moral rationale, ensures AI remains an aid, not an authority. As Aharoni et al. (2024) suggest, people trust AI more when it shares their values and supports their ethical agency. Empowering users to justify decisions themselves also addresses concerns about moral deference.

Policy implications include the need for oversight and accountability mechanisms in AI-driven moral advice. Since cadets were influenced by SIEMA’s recommendations, any AI in decision chains could similarly sway life-or-death outcomes. Military policy should mandate human review or ethics oversight for AI-informed decisions, especially those involving lethal force or non-combatants. Training programs should prepare operators to critically engage AI advice without overreliance, using real-world cases where AI input proved flawed. Transparency policies should require AI systems to explain recommendations, allowing human decision-makers to assess advice against qualitative factors. Hard-coded constraints ensuring respect for ethical limits could further safeguard outcomes (Aharoni et al., 2024; Guzmán et al., 2022). Finally, governance frameworks must clarify responsibility when AI-informed decisions are made to promote accountability while acknowledging the role of AI.

### 5.2 Limitations and future research

Several limitations temper our conclusions. Our sample consisted of military cadets in a specific educational context including training specific to ethical decision-making, which may limit generalizability to other populations and domains. The moral dilemma involved only one type of trade-off, and the content of the recommendations was fixed. Future work should explore a wider range of dilemmas, including those emphasizing values beyond lives saved, and vary the framing and tone of advice. The SIEMA in our study delivered textual recommendations; spoken or embodied communication might produce different effects. Longitudinal studies where advice is repeated across multiple dilemmas could reveal whether people eventually internalize an advisor’s moral orientation. Similar research might also help explain the plasticity of moral judgments observed in this study (e.g., whether participants develop a distinctly “moral” form of trust in robot advisors, rather than merely exhibit the kind of susceptibility to cognitive influence previously identified in HRI research). Finally, although we used utilitarian and deontological labels as convenient descriptors, our study design assumed an inherently consequentialist model of moral judgment –i.e., alternative judgments were presented solely in terms of preferred (expected) outcomes. An important limitation of this assumption is that our results do not decisively indicate whether changes in the preferred outcomes of participants reflect development (“moral learning”) in participants’ moral sensitivities, values, principles, or commitments, or rather simply reflect the influence of a trusted advisor in selecting from alternative outcomes. Future research may leverage non-consequentialist (e.g., deontological or virtue-ethical) approaches to moral judgment by reframing the possible moral judgments available to study participants in terms of rights, principles, fitting emotions, specific virtues, and responsibility.

## 6 Conclusion

This study provides empirical evidence that robotic teammates can influence human moral judgments as effectively as human advisors even after prolonged collaboration. Advice that emphasizes maximizing overall benefit or protecting a particular group temporarily shifts choices toward the recommended direction, but people often rebound toward their initial preferences when presented with alternative input. Qualitative analyses reveal that participants draw on outcomes, duties, trust and personal values when explaining their decisions. These findings highlight both the potential and the limitations of robot-mediated moral persuasion. AI advisors may help humans consider a broader range of ethical perspectives, but lasting change in moral priorities is unlikely to arise from isolated recommendations. As artificial agents play an expanding role in high-stakes decision making, ensuring that they support rather than supplant human moral agency will be essential.

## Data Availability

The raw data supporting the conclusions of this article will be made available by the authors, without undue reservation.
